# Ruptured Superior Mesenteric Artery Pseudoaneurysm With Hemorrhagic Shock Secondary to Infectious Colitis: A Case Report

**DOI:** 10.7759/cureus.103271

**Published:** 2026-02-09

**Authors:** Maha Khan, Emily Erdman, Eric H Chou

**Affiliations:** 1 Surgery, Anne Burnett Marion School of Medicine at Texas Christian University, Fort Worth, USA; 2 Emergency Medicine, Baylor Scott & White Medical Center, Fort Worth, USA; 3 Emergency Medicine, Taipei Hospital, Ministry of Health and Welfare, New Taipei City, TWN

**Keywords:** computed tomography angiography, emergency medicine, hemorrhagic shock, infectious colitis, superior mesenteric artery pseudoaneurysm

## Abstract

Superior mesenteric artery (SMA) pseudoaneurysms are rare, life-threatening vascular abnormalities most often linked to trauma, infection, or iatrogenic injury. Rupture can cause massive hemorrhage and bowel hypoperfusion. We report a unique case of SMA pseudoaneurysm rupture in the setting of infectious colitis, complicated by secondary ischemic colitis. A 66-year-old female with a history of diverticulitis initially presented to the emergency department with abdominal pain and diarrhea, and CT imaging demonstrated non-specific colitis. She was discharged in stable condition with outpatient supportive therapy, but returned three days later with worsening symptoms and hemodynamic instability. CT angiography revealed a ruptured SMA pseudoaneurysm with active intraperitoneal bleeding. Urgent transcatheter embolization was performed, but she subsequently developed bowel ischemia and perforation requiring multiple operations, including right colectomy and small bowel resection. Following a prolonged ICU course, she was ultimately discharged to long-term acute care in stable condition. This case highlights an unusual progression from infectious colitis to vascular catastrophe, followed by ischemic colitis. Early recognition of the potential for septic versus hemorrhagic shock in infectious colitis with hypotension, along with CT angiography and coordinated multidisciplinary management, was critical. Clinicians should maintain suspicion for vascular complications in colitis patients who present with hemorrhage or shock, as early diagnosis and intervention may prevent catastrophic outcomes.

## Introduction

Infectious colitis is inflammation of the colon’s mucosal lining, acute or chronic, which is common and increasingly prevalent worldwide [1]. Although infectious colitis affects all age groups, severe cases in older patients could lead to complications such as gastrointestinal hemorrhage or less common extra-intestinal inflammatory sequelae [2]. Severe colitis can evolve into toxic colitis or megacolon when associated with bowel dilation and systemic manifestations, resulting in life-threatening outcomes [2]. Patients may present with disproportionate abdominal pain relative to modest peritoneal exam findings. Typical features include tenderness to palpation, diarrhea, distension, bloody stools, and, sometimes, postprandial pain [3]. Infectious colitis is associated with intense mucosal inflammation, which may disrupt the integrity of adjacent mesenteric tissues. In severe cases, this inflammatory process could extend to nearby mesenteric arterial branches, resulting in arterial wall injury or increased vulnerability [4]. This inflammatory arterial injury could then lead to a fragile pseudoaneurysm lacking normal media, predisposing to rupture or even spontaneous rupture [5].

Superior mesenteric artery (SMA) involvement accounts for approximately 8-10% of visceral artery aneurysms overall, a category that includes both true aneurysms and pseudoaneurysms [6]. Visceral artery pseudoaneurysms, however, represent a distinct entity and are more commonly associated with trauma, infection, pancreatitis, iatrogenic injury, or regional inflammation [7]. While the precise anatomic distribution of pseudoaneurysms may differ from that of true aneurysms, SMA pseudoaneurysms remain rare and clinically significant due to their high propensity for rupture. Reported mortality rates following rupture of visceral artery pseudoaneurysms range from 40% to 60%, and approximately 22% of patients require emergent intervention, underscoring the urgency of prompt diagnosis and management [8,9]. Although uncommon, several cases describing catastrophic rupture have been reported in patients presenting with severe abdominal pain or hemodynamic instability [10].

To our knowledge, cases describing SMA pseudoaneurysm rupture occurring in the setting of infectious colitis are rarely reported in the literature. Clinicians should consider occult mesenteric pseudoaneurysm in patients with colitis who fail to improve clinically or who develop unexplained recurrent abdominal pain, bleeding, or signs of hemodynamic instability. This case illustrates a potential association between presumed infectious colitis and subsequent mesenteric vascular catastrophe, despite limited prior evidence directly linking these entities managed with endovascular embolization and surgical resection, followed by secondary ischemic colitis. This experience demonstrates the importance of early arterial-phase vascular imaging and coordinated interventional and surgical management when a patient with colitis acutely deteriorates.

## Case presentation

A 66-year-old female with a medical history of hypertension and diverticulitis presented to the emergency department with a 10-day history of fluctuating abdominal pain, associated with nausea, vomiting, and diarrhea. She denied anticoagulant use. On arrival, her vital signs were stable, and the physical examination was unremarkable, without abdominal distension or guarding. Laboratory results were unremarkable except for mild leukocytosis. Computed tomography (CT) of the abdomen and pelvis showed mural thickening of the ascending colon and cecum, along with moderate thickening of the transverse, descending, and sigmoid colon, consistent with nonspecific colitis. Given her stable hemodynamic status, absence of peritoneal signs, and lack of alarming features on initial evaluation, she was discharged with outpatient supportive therapy, including oral hydration, symptomatic management with antidiarrheal and antiemetic medications, and strict return precautions. No stool studies, blood cultures, or *Clostridioides difficile* testing were performed at the initial visit, and the diagnosis of acute colitis was presumed based on clinical presentation and imaging findings.

**Figure 1:**
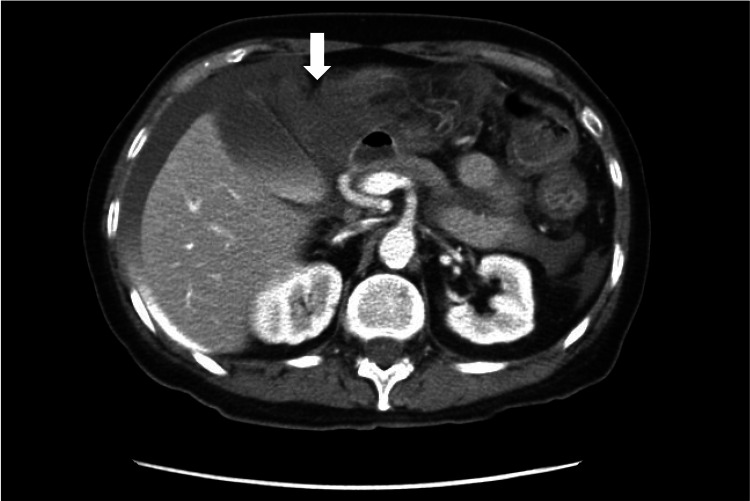
Axial contrast-enhanced CT of the abdomen demonstrating active contrast extravasation consistent with hemoperitoneum (arrow).

**Figure 2:**
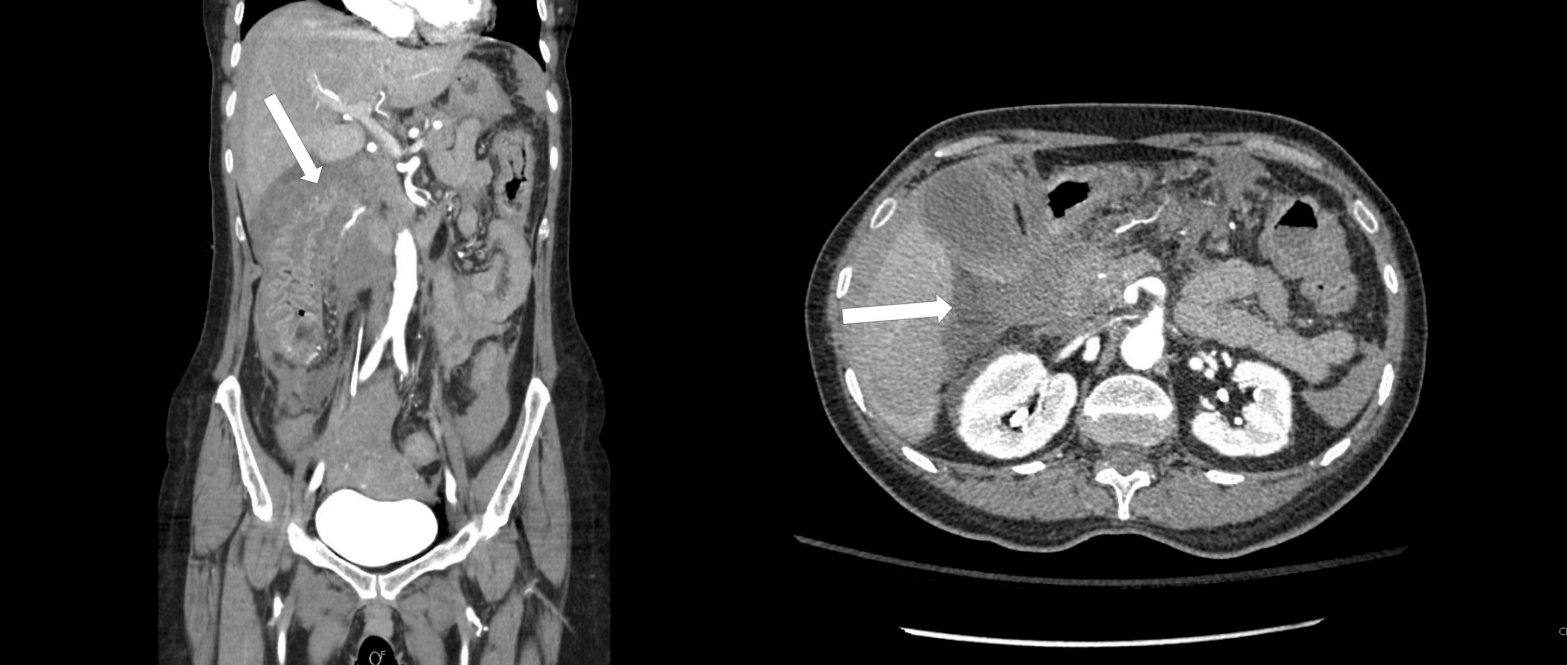
Axial CT angiography of the abdomen and pelvis demonstrating a pseudoaneurysm with active contrast extravasation from a mesenteric branch of the superior mesenteric artery (arrow).

She returned three days later with recurrent abdominal pain accompanied by nausea, vomiting, and diarrhea. On arrival, she was afebrile but visibly distressed due to pain, with an initial blood pressure of 176/90 mmHg and no evidence of hypotension. Initial laboratory studies demonstrated worsening leukocytosis and a mildly elevated lactate level (Table 1), raising concern for sepsis in the setting of presumed infectious colitis. She was started on intravenous fluids, broad-spectrum antibiotics (piperacillin-tazobactam), and analgesia. A repeat contrast-enhanced CT scan of the abdomen and pelvis revealed worsening pancolitis with new right-sided hemoperitoneum and evidence of active contrast extravasation, without free intraperitoneal air (Figure 1). Shortly thereafter, serial laboratory testing showed a rapid decline in hemoglobin levels and rising lactate, shifting concern from isolated sepsis to hemorrhagic shock (Table 1). Subsequent CT angiography of the abdomen and pelvis identified an 8.5 × 11 mm pseudoaneurysm arising from a branch of the superior mesenteric artery, with associated hemoperitoneum extending into the pelvis and an adjacent mesenteric hematoma (Figure 2).

**Table 1:** Summary of key laboratory tests performed during the first emergency department (ED) visit, at presentation during the second ED visit, and during early clinical deterioration after the second ED visit. Absolute laboratory values are listed with units to show trends over time. “NA” indicates tests that were not performed at that time point.

Laboratory parameter	1st ED visit	2^nd^ ED visit	2^nd^ ED visit follow-up (after 5 hours)	Reference range	ED course	Clinical trend
White blood cell count (×10³/µL)	15.7	31.3	21.6	4.0–10.0	Increased	Progressive leukocytosis
Hemoglobin (g/dL)	13.7	14.0	5.0	12.0–16.0	Decreased	Acute blood loss
Hematocrit (%)	41.0	42.1	15.1	36–46	Decreased	Acute blood loss
Serum lactate (mmol/L)	NA	1.9	5.1	0.5–2.0	Increased	Tissue hypoperfusion
Platelet count (×10³/µL)	302	321	206	150–400	Decreased	Consumptive coagulopathy
INR	1.0	NA	1.4	0.9–1.1	Increased	Coagulopathy
Serum creatinine (mg/dL)	1.0	0.89	1.07	0.6–1.2	Increased	Acute kidney injury

During this period, the patient developed hemodynamic instability with hypotension (69/58 mmHg) and experienced a brief pulseless episode requiring resuscitation, raising concern for septic versus hemorrhagic shock. Given the evidence of active hemorrhage, acute anemia, and unstable vital signs, she was emergently transferred to interventional radiology for definitive hemorrhage control. Catheter-based angiography showed active bleeding from two branches of the middle colic artery. These vessels were treated with selective coil embolization until stasis was reached. Additionally, angiographic irregularity of a branch of the right colic artery was identified and treated with gel foam embolization. Completion angiography confirmed the cessation of active contrast extravasation from the treated vessels. The patient received four units of packed red blood cells during resuscitation along with electrolyte correction. No anticoagulation reversal was needed, as she was not on anticoagulant therapy at baseline. Vasopressor support was used briefly during resuscitation due to hemodynamic instability. She tolerated the procedure without immediate complications and was admitted to the intensive care unit for close monitoring.

**Figure 3:**
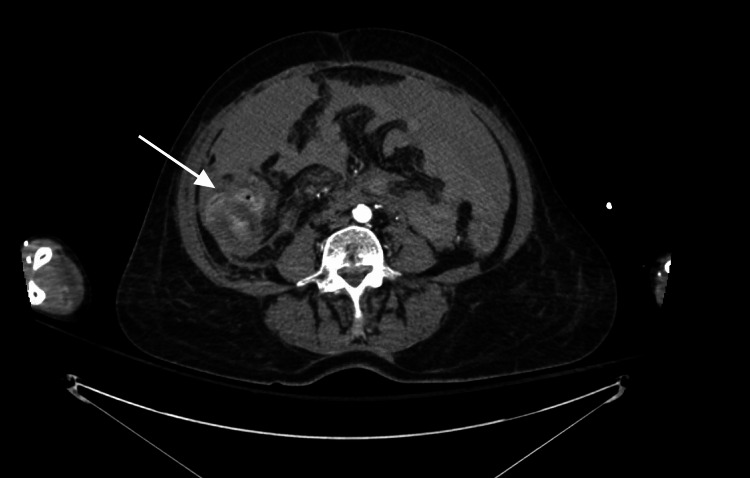
A repeat CT scan demonstrated ongoing active contrast extravasation into the right peritoneal cavity and the lumen of the ascending colon, along with progressive hemoperitoneum (arrow).

On postoperative day one, her hemoglobin decreased again, and she was transfused with multiple blood products as part of her resuscitation. A repeat CT scan demonstrated ongoing active contrast extravasation into the right peritoneal cavity and the lumen of the ascending colon, along with progressive hemoperitoneum (Figure 3). General and vascular surgery were consulted. Despite imaging evidence of ongoing extravasation, the patient remained hemodynamically stable following transfusion, with no signs of peritonitis, bowel ischemia, or recurrent hemodynamic compromise. Given her recent hemorrhagic shock, transient cardiac arrest, and high operative risk, the decision was made to pursue continued nonoperative management rather than immediate surgical exploration or repeat embolization. Repeat endovascular intervention was also deferred due to concern for further mesenteric ischemia in the setting of recent embolization and extensive colitis. Conservative management included continuous hemodynamic monitoring, serial abdominal examinations, and close laboratory surveillance with hemoglobin and lactate measurements. Enteral nutrition was initiated as trickle feeds via nasogastric tube to assess bowel tolerance.

**Figure 4:**
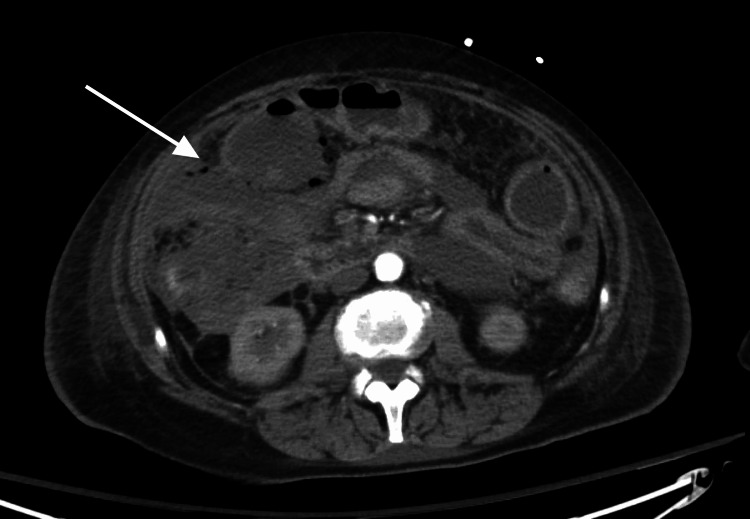
CT imaging demonstrated hydropneumoperitoneum, pneumatosis intestinalis, and bowel wall thickening, findings consistent with bowel ischemia and perforated viscus (arrow).

On postoperative day 12, the patient developed worsening respiratory distress, marked leukocytosis, acute kidney injury, and progressive abdominal distension. CT imaging demonstrated hydropneumoperitoneum, pneumatosis intestinalis, and bowel wall thickening, findings consistent with bowel ischemia and perforated viscus (Figure 4). Her clinical exam deteriorated with significant abdominal distension, guarding, worsening leukocytosis, and rising lactate, prompting emergent surgical intervention.

Exploratory laparotomy revealed a large volume of hemoperitoneum, extensive necrosis of the right colon, two perforations in the proximal transverse colon, and multiple noncontiguous segments of necrotic ileum with associated perforations. Ischemic injury involved both colonic and small bowel segments and was not confined to a single vascular distribution. Mesenteric pulses were present in uninvolved segments, and the pattern of injury suggested global hypoperfusion rather than focal arterial occlusion. Surgical management included evacuation of the hemoperitoneum, right colectomy, small bowel resection, and placement of an ABThera negative pressure wound therapy device. Given the extent of ischemia and physiologic instability, the bowel was left in discontinuity with plans for staged re-exploration.

Histologic examination demonstrated severe acute colitis with transmural ischemic necrosis extending into the pericolonic fibroadipose tissue. No histopathologic features of chronic inflammatory bowel disease or primary vasculitis were identified. The ischemic injury involved both small and large bowel segments and extended beyond a single embolized vascular territory, arguing against isolated embolization-related ischemia. Instead, the distribution supports secondary ischemic injury due to hemorrhagic shock in the setting of severe colonic inflammation. Taken together, these findings are most consistent with a ruptured superior mesenteric artery pseudoaneurysm causing hemorrhagic shock in a patient initially presenting with presumed infectious colitis complicated by sepsis.

She returned to the operating room 48 hours later for re-exploration, abdominal washout, and jejunal-colonic anastomosis. No further evidence of ischemia or hemorrhage was identified. Five days after abdominal closure, she developed bilious emesis and suspected aspiration pneumonitis, resulting in respiratory distress and necessitating re-intubation. Her nutrition was supported with total parenteral nutrition until she was able to advance to tube feeds. The patient was subsequently downgraded from ICU level of care and transferred to a long-term acute care facility in stable condition. No additional complications were observed within 30 days. A comprehensive timeline of events from her initial presentation through disposition is shown in Table 2.

**Table 2:** Chronological summary of the patient’s clinical course from initial presentation to disposition. CTA: computed tomography angiography; SMA: superior mesenteric artery; PEA: pulseless electrical activity; ROSC: return of spontaneous circulation; IR: interventional radiology; PRBC: packed red blood cells; NG: nasogastric.

Time point	Key findings	Management/outcome
Day 0: Initial ED visit	10 days of abdominal pain, nausea, vomiting, diarrhea; vitals stable, CT showed nonspecific pancolitis.	Discharged with outpatient supportive treatment
Day 3: Return to ED	Worsening abdominal pain, leukocytosis, and lactate elevation. CT: pancolitis with hemoperitoneum; CTA: SMA branch pseudoaneurysm with active extravasation. Became hypotensive with PEA arrest. ROSC after resuscitation.	IR coil embolization of middle colic branches with Gelfoam; 4 units PRBC transfused; ICU admission
Days 4–11: Post-embolization	Persistent anemia and coagulopathy. Repeat CT showed ongoing extravasation.	Managed conservatively with NG trickle feeds and close monitoring
Days 12–13: Acute deterioration	Marked leukocytosis, acute kidney injury, and respiratory distress. CT: pneumatosis intestinalis and hydropneumoperitoneum.	Emergent exploratory laparotomy
Day 14: Second-look laparotomy	No further hemorrhage or ischemia identified.	Abdominal washout and jejunal–colonic anastomosis
Days 15–20: Post-operative course	Aspiration pneumonitis requiring re-intubation; ileus.	Conservative management; total parenteral nutrition
Day 21: Disposition	Clinical stabilization; follow-up CT stable at 2 months.	Transferred to long-term acute care facility

## Discussion

Visceral artery pseudoaneurysms (VAPAs) are rare but highly lethal vascular lesions, most commonly involving the splenic, hepatic, and superior mesenteric arteries. Although uncommon, rupture carries a reported mortality of up to 75%, warranting prompt diagnosis and intervention even in asymptomatic patients. Known etiologies include trauma, infection, inflammation, pancreatitis, atherosclerosis, and iatrogenic injury. SMA pseudoaneurysms, in particular, pose a diagnostic challenge due to their rarity and nonspecific clinical presentation [11,12]. This case is unique in that the patient developed a ruptured SMA branch pseudoaneurysm following an episode of infectious colitis without traditional precipitating risk factors such as trauma, pancreatitis, anticoagulation, or recent surgery. We hypothesize that severe localized colonic inflammation led to extension of the inflammatory process to adjacent mesenteric arterial branches, compromising vessel integrity and predisposing to pseudoaneurysm formation. Although causation cannot be definitively established in the absence of histopathologic vascular analysis, this case suggests a previously underrecognized association between severe colitis and mesenteric arterial injury.

Ischemic colitis results from either occlusive or non-occlusive compromise of colonic blood flow [11,12]. Occlusive mechanisms include arterial thrombosis, embolism, or mechanical obstruction, while nonocclusive ischemia is often related to systemic hypoperfusion. Inflammatory-mediated vascular injury represents a less commonly described mechanism. In our patient, the acute infectious colitis likely caused significant localized inflammation that extended to branches of the superior mesenteric artery. Her past medical history of hypertension and diverticulitis suggests her arterial vessels may have already been compromised or predisposed to inflammatory injury. This inflammatory response, possibly combined with infectious mediators, could have weakened the integrity of SMA branches, rendering them vulnerable to pseudoaneurysm formation. In this patient, early imaging demonstrated nonspecific colitis, likely reflecting early ischemia or inflammatory insult. Rapid progression to vascular rupture highlights the dynamic and potentially catastrophic nature of mesenteric vascular compromise.

Endovascular therapy has emerged as the preferred first-line treatment for visceral pseudoaneurysms due to lower morbidity, faster hemorrhage control, and avoidance of general anesthesia [13]. Coil embolization, in particular, offers excellent short- and long-term outcomes and was appropriately selected in this hemodynamically unstable patient [13,14]. Surgical intervention remains reserved for cases involving infected pseudoaneurysms, failure of endovascular therapy, or complications such as bowel necrosis, all of which ultimately occurred in this patient. Her subsequent ischemic bowel requiring surgical resection reflects a known limitation of embolization, namely, the risk of downstream ischemia. According to the American College of Gastroenterology, while surgical repair can be performed for a ruptured pseudoaneurysm, coil embolization also provides excellent results and is recommended due to its less invasive nature [15,16].

It is important to contextualize this case within current literature on VAPAs and their known associations, considering the rarity of this complication. Visceral artery aneurysm (VAA) most frequently affects the splenic (60%), hepatic (20%), and superior mesenteric arteries (9%), with 11 cases reported of a traumatic pseudoaneurysm after a motor vehicle collision [17]. Three case reports describe an SMA mycotic pseudoaneurysm following infective endocarditis (IE) in a patient with rheumatic heart disease, most likely due to bacterial thromboses causing infarction of the affected vessel [18,19]. This highlights a gap in the literature and explains why our case differs from previously reported causes [18-20]. Unlike what is described in current papers, our patient had no identifiable risk factors for the pseudoaneurysm, such as trauma, surgery, pancreatitis, anticoagulation, or iatrogenic injury. This case expands the known causes of SMA pseudoaneurysms. It highlights that severe colonic inflammation alone can compromise nearby blood vessels, underscoring the importance of considering CTA in patients with colitis who experience sudden worsening without an apparent reason. This case is the first to raise the possibility that severe colonic inflammation may predispose to mesenteric arterial injury; however, the absence of histopathologic findings and a detailed examination in this regard limits our ability to establish the first causal relationship. Therefore, this clinical case should be regarded as an association rather than definitive evidence of causation and should be noted as such rather than a proven causal mechanism.

Regarding the treatment of visceral pseudoaneurysms, surgical treatment was initially the first choice of treatment, including bypass procedure with resection, arterial ligation, and visceral organ resection. However, the literature has demonstrated time and again that surgery for visceral pseudoaneurysm has disadvantages such as a long hospitalization time, requirement of general anesthesia, and poor wound healing in those with comorbidities, along with a higher morbidity and mortality rate compared to endovascular treatment [18-20]. Therefore, interventional radiological treatment, including endovascular approaches, has taken first place in the treatment of pseudoaneurysms; however, surgery remains the gold standard for those with infected pseudoaneurysms, rapid growth, ischemia, neuropathy, or failure of endovascular treatment. In our case, embolization was likely chosen as a first-line treatment, given its good short- and long-term results, complete symptom resolution, and, theoretically, no risk of continued infection. It is also essential to consider the patient’s acute presentation with hemodynamic instability and at high operative risk, considering her medical history, which meant that embolization was the fastest means of achieving hemorrhage control with lower physiologic compromise. This decision-making is supported by current vascular guidelines, which favor embolization as a first-line therapy as described above. The only limitation of endovascular techniques is challenging target vessel access, arterial tortuosity, the need to conserve side branches at or near the repair site, and even endovascular failure, which can make complete endovascular repairs difficult and was likely why surgery was warranted nearly two weeks after embolization in this case for the ischemic bowel [19,20].

Distinguishing septic from hemorrhagic shock in real time was challenging in this patient. Leukocytosis, abdominal pain, and elevated lactate initially raised concern for sepsis; however, the rapid decline in hemoglobin, presence of hemoperitoneum, and transient pulseless episode favored hemorrhagic shock as the primary etiology. Accordingly, the differential diagnosis appropriately prioritized gastrointestinal bleeding and vascular causes, particularly given her recent history of ischemic colitis, while spontaneous bleeding related to anticoagulation was considered less likely. This case highlights the importance of maintaining diagnostic vigilance for both septic and hemorrhagic shock in patients with colitis and acute hemodynamic deterioration, as delayed recognition of hemorrhagic shock may result in catastrophic outcomes.

## Conclusions

SMA pseudoaneurysms are rare but potentially life-threatening, most often associated with trauma, surgery, pancreatitis, or iatrogenic injury. In this case, rupture occurred in the setting of presumed infectious colitis complicated by sepsis. Prompt CT angiography was critical for diagnosis in the setting of sudden clinical deterioration. Successful management with endovascular embolization followed by surgical resection for secondary ischemic injury highlights the importance of early vascular imaging and a multidisciplinary approach. Although causality cannot be definitively established, this case adds to the limited literature describing SMA pseudoaneurysm rupture associated with severe acute colitis and broadens understanding of potential etiologies.
